# The histone demethylase LSD1 promotes renal inflammation by mediating TLR4 signaling in hepatitis B virus-associated glomerulonephritis

**DOI:** 10.1038/s41419-019-1514-4

**Published:** 2019-03-20

**Authors:** Yi-Tong Yang, Xuan Wang, Yue-Yue Zhang, Wei-Jie Yuan

**Affiliations:** Department of Nephrology, Shanghai General Hospital, Shanghai Jiao Tong University School of Medicine, Shanghai, 200080 China

## Abstract

Renal inflammation significantly contributes to the progression of hepatitis B virus (HBV)-associated glomerulonephritis (HBV-GN), but the mechanisms that control its precise regulation remain largely unknown. In this study, we showed that the lysine-specific demethylase 1 (LSD1) was significantly upregulated in renal tissue of HBV-GN patients, and its expression was positively correlated with inflammation. Functionally, LSD1 could promote HBV-induced release of proinflammatory mediators in HK-2 cells, a human renal tubular epithelial (RTE) cell line. Mechanistic investigations suggested that LSD1 directly promoted the transcription of the inflammatory-related gene *Tlr4* by eliminating the mono- or di-methylation of H3K9 near its promoter. Knockdown of *Lsd1* further inhibited TLR4-NF-κB/JNK signaling cascades, and subsequently decreased HBV-induced production of proinflammatory mediators in HK-2 cells. Co-transfection with *Tlr4*-expressing plasmids counteracted these effects. Meanwhile, downregulation of abovementioned TLR4-related pathways using small-molecule inhibitors attenuated inflammation. Importantly, LSD1 inhibitor tranylcypromine (TCP) could inhibit TLR4-NF-κB/JNK signaling axis and alleviate renal inflammation in HBV transgenic mice. Taken together, our data identify LSD1 as a novel regulator of renal inflammation and as a potential therapeutic target in HBV-GN.

## Introduction

Persistent infection with hepatitis B virus (HBV) can result in HBV-associated glomerulonephritis (HBV-GN), which has become one of the major secondary renal diseases in China^1^. HBV-GN is generally believed to be caused by immune complex deposition^2^, but several studies have identified expression of HBV antigens in the kidneys and revealed that virus replication and direct virus-induced pathological alterations are also involved^3,4^. Our earlier studies have found inflammatory cells infiltration and tubulointerstitial injury in the renal biopsies from HBV-GN patients^5,6^, suggesting that local inflammation might also be involved in this disease process. Similarly, other studies have also shown that renal inflammation induced by HBV could cause renal injury contributing to the progression of HBV-GN^7,8^. Therefore, renal inflammation was thought to be a potential target for attenuating HBV-GN. However, the molecular mechanisms that regulate it remain unclear.

Epigenetic modifications, mainly including genomic DNA methylation and histone modifications, have been shown to play a vital role in the regulation of renal inflammation^9–11^. These modifications can change and influence the accessibility for transcription factor binding, thereby regulating gene transcription and cellular functions^12–15^. There are several histone modifications, including acetylation, methylation, phosphorylation, ubiquitination, and sumoylation^16^. A recent study has demonstrated that histone acetylation could contribute to the exacerbation of renal inflammation^17^. However, the role of other histone modifications, particularly histone methylation, in the regulation of the above process remains unknown.

It has become increasingly clear that histone methylation, unlike acetylation, does not alter the lysine charge but changes transcription by providing docking sites for chromatin modifiers^16^. Lysine residues of histone proteins can be mono-, di-, and tri-methylated, which is regulated by both histone lysine methyltransferases and lysine demethylases. The histone lysine-specific demethylase 1 (LSD1), a flavin-containing amino oxidase, specifically removes both the mono- or di-methylation of histone 3 lysine 4 (H3K4me1/2) and histone 3 lysine 9 (H3K9me1/2)^18,19^. According to previous studies, H3K4me1/2 is generally associated with transcriptionally active genes, whereas H3K9me1/2 is associated with transcription silencing^20–23^. LSD1 has been involved in wide-ranging biological processes, including cell proliferation^24^, chromosome segregation^25^, hematopoiesis^26^, spermatogenesis^27^, adipogenesis^28^, stem cell pluripotency^29^, and embryonic development^30^. LSD1 also acts as an oncogene, and its overexpression promotes cancer cell proliferation, migration, and invasion^31,32^. In addition, LSD1 has been identified as a critical epigenetic regulator of the inflammatory response in sepsis^33^. However, little is known about its precise role in the renal inflammation in HBV-GN.

In the present study, we explored the potential effects and the underlying mechanisms of LSD1 on renal inflammation in HBV-GN. Our results demonstrated that LSD1 could promote HBV-induced production of proinflammatory mediators in vitro by epigenetically upregulating the expression of toll-like receptor 4 (TLR4), an important receptor of innate and acquired immunity^34^, thereby contributing to the activation of nuclear factor-κB (NF-κB) and JNK pathways. Together, these findings provide a novel insight into the underlying mechanisms of renal inflammation induced by HBV in HBV-GN.

## Results

### LSD1 is significantly upregulated and positively correlated with inflammation in renal tissue of HBV-GN

**Fig. 1:**
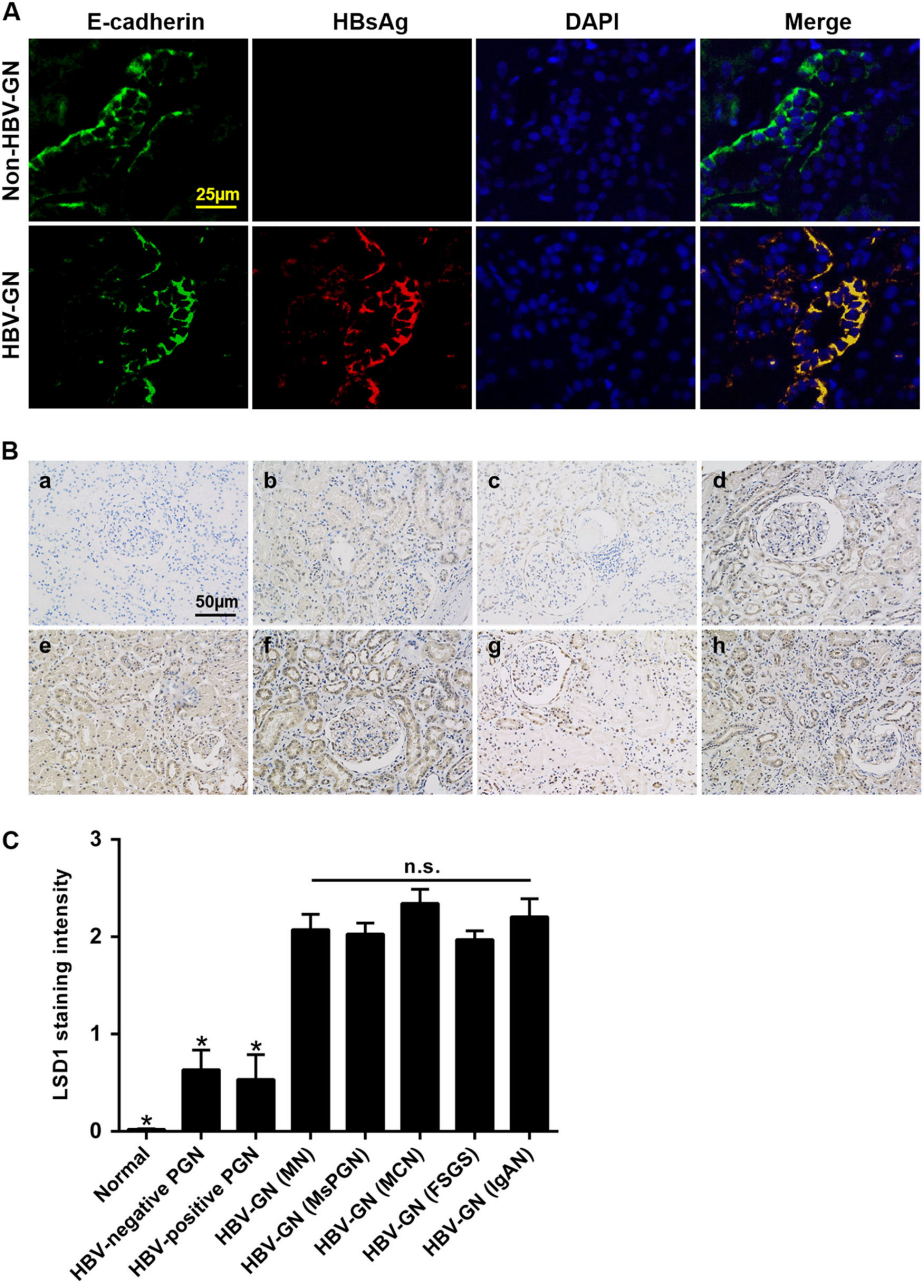
Elevated lysine-specific demethylase 1 (LSD1) expression levels in patients with hepatitis B virus-associated glomerulonephritis (HBV-GN). **a** Immunofluorescence for HBsAg and E-cadherin in HBV-GN and non-HBV-GN groups. E-cadherin was used as a tubule epithelial marker. **b** Immunohistochemistry for LSD1 in HBV-GN group and other pathological conditions. a Normal control group, b HBV-negative primary glomerulonephritis (PGN) group, c HBV-positive PGN group, d membranous nephropathy (MN), e mesangial-proliferative glomerulonephritis (MsPGN), f minimal change nephropathy (MCN), g focal segmental glomerulosclerosis (FSGS), and h IgA nephropathy (IgAN). d–h The different pathology types for HBV-GN group. **c** Mean LSD1 staining intensity in each group. Staining intensity was graded on a scale of 0 (no staining) to 3+ (intense staining). Data are expressed as the mean ± SD. *P < 0.05 versus HBV-GN. n.s. not significant

There were no significant differences in the general, clinical features, renal pathology (glomerular and tubular scores), and liver function of patients in the HBV-GN, HBV-positive, and -negative primary glomerulonephritis (PGN) groups (Supplementary Table S1). All 53 patients in the HBV-GN group were positive for HBsAg in serum and renal tissue (Supplementary Table S1 and Fig. 1a). In the normal control group, LSD1 could not be obviously detected by immunohistochemistry (IHC). However, the expression of LSD1 was observed in various pathological types of HBV-GN group (Fig. 1b). LSD1 was mainly deposited in the renal tubular epithelial (RTE) cells and was also detected in the glomerulus (Fig. 1b). A semiquantitative analysis of staining intensity demonstrated higher expression for LSD1 in HBV-GN group than in non-HBV-GN groups (Fig. 1c). Meanwhile, a comparison of LSD1 staining intensity among various pathological types in HBV-GN group showed no significant difference (Fig. 1c). Analysis of immunohistochemical staining of LSD1 revealed that there was a positive correlation between LSD1-positive staining score and the tubular pathology score (*r* = 0.702, *P* < 0.001), tubular atrophy (*r* = 0.673, *P* < 0.001), and interstitial fibrosis (*r* = 0.596, *P* < 0.001) in the HBV-GN group.

**Fig. 2:**
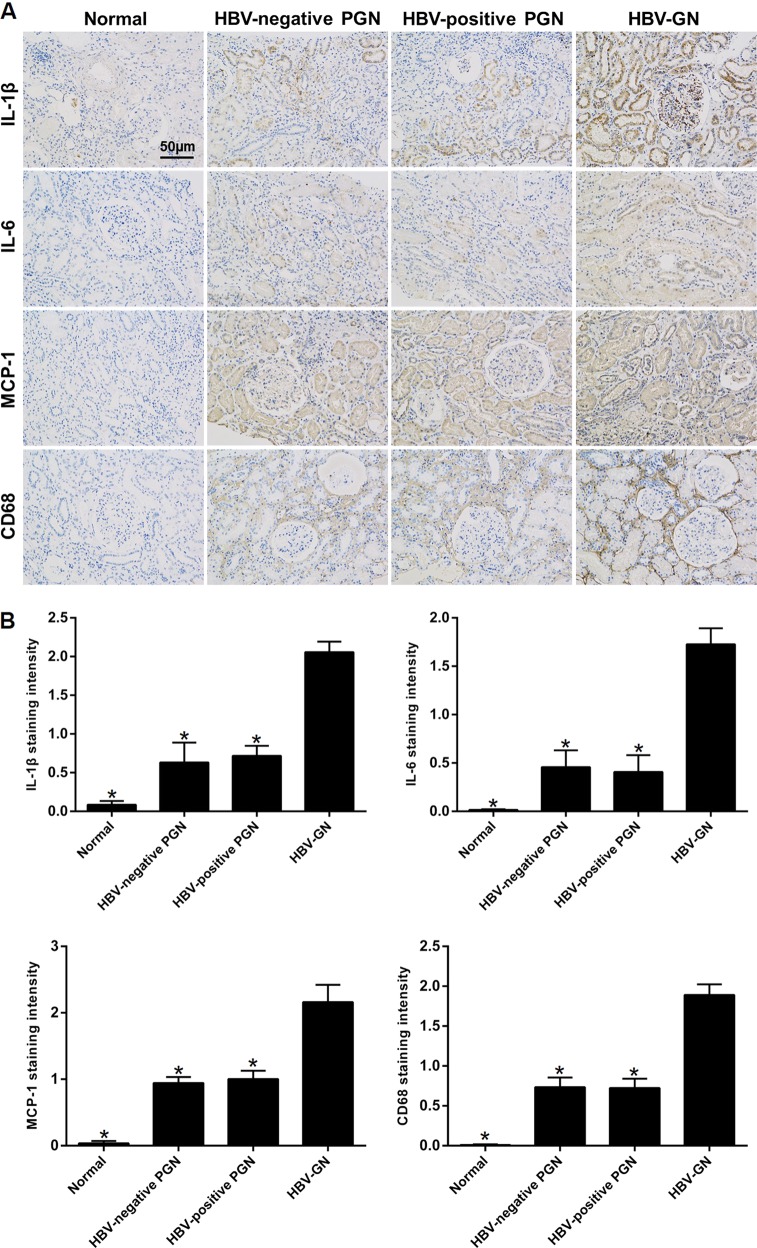
Increased inflammatory responses in renal tissues of hepatitis B virus-associated glomerulonephritis (HBV-GN). **a** Immunohistochemistry for interleukin (IL)-1β, IL-6, monocyte chemoattractant protein-1 (MCP-1), and CD68 in HBV-GN and non-HBV-GN groups. **b** Mean IL-1β, IL-6, MCP-1, and CD68 staining intensity in each group. Staining intensity was graded as indicated in Fig. 1c. Data are expressed as the mean ± SD. *P < 0.05 versus HBV-GN

In our previous study, IHC analysis in the renal biopsies showed that the expression of tumor necrosis factor-alpha (TNF-α) was increased in HBV-GN^35^. In the present study, IHC analysis in the renal biopsies also showed that the expression of other inflammatory markers, including interleukin (IL)-1β, IL-6, monocyte chemoattractant protein-1 (MCP-1), and CD68 (macrophage marker), was markedly increased in HBV-GN than in non-HBV-GN (Fig. 2a, b). These results revealed that inflammatory responses might be involved in this disease process. Previous studies have reported that LSD1 could regulate the expression of IL-1β and IL-6 in other diseases^36,37^. Hence, it was reasonable to hypothesize that there might be a positive correlation between the expression of LSD1 and above inflammatory markers in renal tissue of HBV-GN. Expectedly, statistical analysis revealed that the expression of LSD1 was positively correlated with that of IL-1β (*r* = 0.716, *P* < 0.001), IL-6 (*r* = 0.684, *P* < 0.001), and MCP-1 (*r* = 0.679, *P* < 0.001) (Table 1). Taken together, these data suggested that LSD1 expression was significantly upregulated and positively correlated with inflammation in renal tissue of HBV-GN, and that upregulation of LSD1 corresponding to HBV infection might contribute to the inflammatory damage associated with the progression of HBV-GN.

**Table 1:** Correlations between the LSD1 expression and the IL-1β, IL-6, or MCP-1 expression in HBV-GN patients

LSD1	IL-1β				IL-6				MCP-1			
	−	+	++	+++	−	+	++	+++	−	+	++	+++
−	3	0	0	0	2	1	0	0	3	0	0	0
+	2	6	0	0	3	4	1	0	1	5	1	1
++	0	2	10	5	3	4	9	1	0	3	9	5
+++	0	1	7	17	1	2	7	15	0	0	7	18
*r*_*s*_	0.716				0.684				0.679			
*P*	<0.001				<0.001				<0.001			

*LSD1* lysine-specific demethylase 1, *IL* interleukin, *MCP-1* monocyte chemoattractant protein-1, *HBV-GN* hepatitis B virus-associated glomerulonephritis

### LSD1 promotes the release of proinflammatory mediators in HBV-infected HK-2 cells

**Fig. 3:**
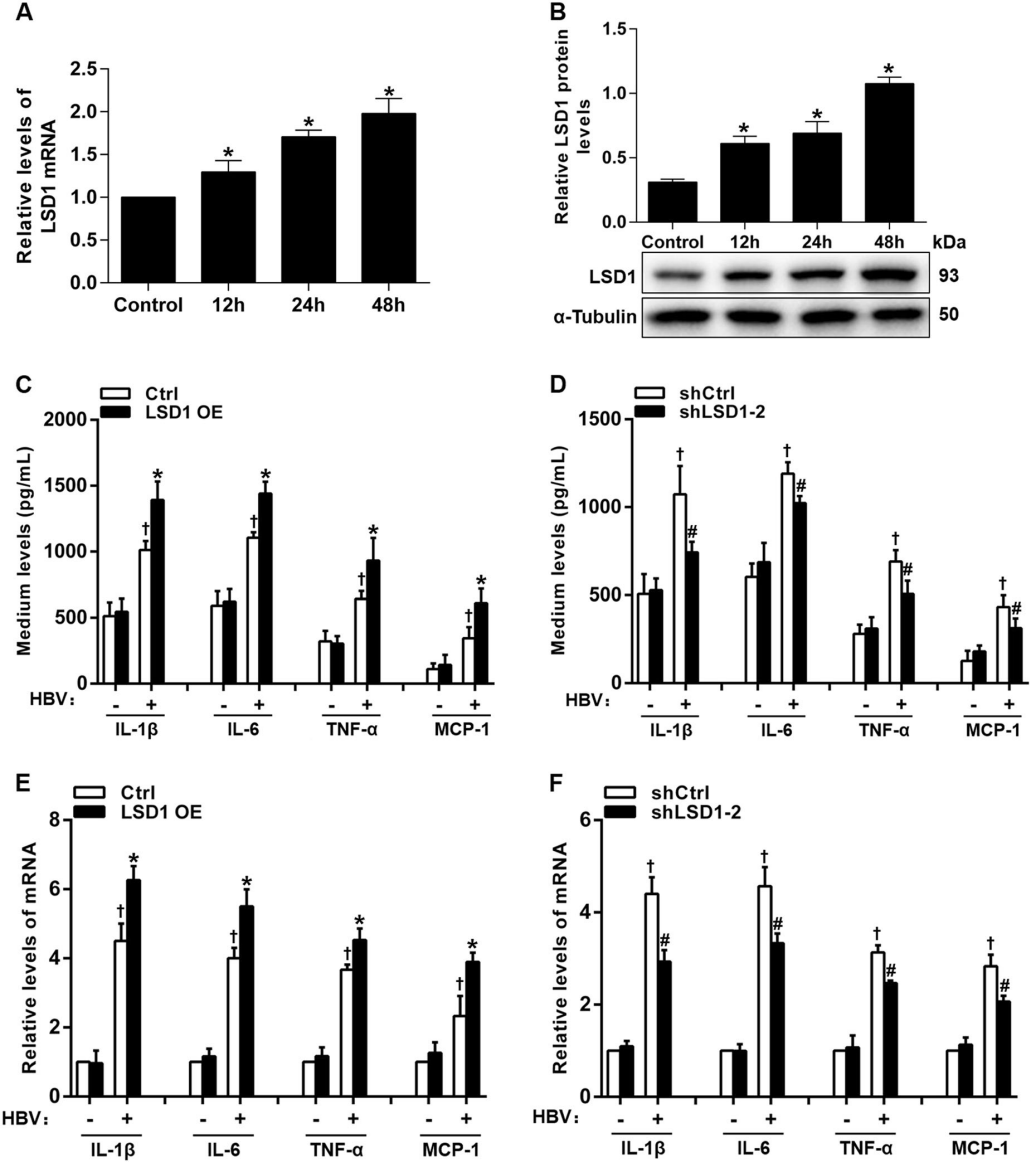
Lysine-specific demethylase 1 (LSD1) increases the production of proinflammatory mediators in hepatitis B virus (HBV)-infected HK-2 cells. **a**, **b** Quantitative real-time PCR (qRT-PCR) and western blot analysis of LSD1 mRNA (**a**) and protein (**b**) in HK-2 cells transfected with pCMV-HBV1.3 at different times. **c**, **d** Enzyme-linked immunosorbent assay analysis of proinflammatory mediators including interleukin (IL)-1β, IL-6, tumor necrosis factor-alpha (TNF-α), and monocyte chemoattractant protein-1 (MCP-1) levels in culture medium of HK-2 cells co-transfected with pCMV-HBV1.3 and LSD1 overexpressed (OE) plasmid (**c**) or shLSD1-2 (**d**) for 48 h. **e**, **f** qRT-PCR analysis of proinflammatory mediators mRNA expression in HK-2 cells co-transfected as indicated in **c** and **d**, respectively. Data are presented as the mean ± SD (*N* = 3). *P < 0.05 versus Ctrl; ^#^*P* < 0.05 versus shCtrl; ^†^*P* < 0.05 versus no HBV infection

To explore the role of LSD1 in regulating the expression of proinflammatory mediators in vitro, first we transfected pCMV-HBV-1.3 plasmid into HK-2 cells, a human RTE cell line, to establish HBV infection model. After transfection for 48 h, the levels of HBsAg and HBeAg in the cell supernatant were detected. The results showed that HBsAg and HBeAg levels in the supernatant of HK-2 cells transfected with pCMV-HBV1.3 remained at a high level, whereas no HBsAg and HBeAg were detected in that of HK-2 cells transfected with empty vector (Supplementary Figure S1A, B). The results indicated that HBV could successfully infect HK-2 cells. Then, we measured *Lsd1* mRNA and protein expression levels at the indicated time points after transfection in HK-2 cells with pCMV-HBV1.3. The results showed that HBV could significantly increase *Lsd1* mRNA and protein levels (Fig. 3a, b). Finally, we confirmed the efficiency of the *Lsd1* knockdown and overexpression protocols in HBV-infected HK-2 cells. The results revealed that *Lsd1* overexpressed plasmid (LSD1 OE) and *Lsd1* shRNAs (shLSD1) could significantly increase and decrease LSD1 protein expression, respectively (Supplementary Figure S2A, B). Quantitative real-time PCR (qRT-PCR) assay and enzyme-linked immunosorbent assay (ELISA) were carried out to detect the levels of proinflammatory mediators in HK-2 cells with different treatments. The results indicated that *Lsd1* overexpression significantly increased the levels of proinflammatory mediators in culture medium of HK-2 cells infected with HBV (Fig. 3c), whereas *Lsd1* knockdown significantly decreased their levels (Fig. 3d). Furthermore, their mRNA expression was significantly promoted by *Lsd1* overexpression (Fig. 3e) and inhibited by *Lsd1* knockdown (Fig. 3f). These data together suggested that LSD1 was able to promote HBV-induced production of proinflammatory mediators in vitro.

### *Tlr4* expression is decreased after *Lsd1* knockdown in HBV-infected cells

**Fig. 4:**
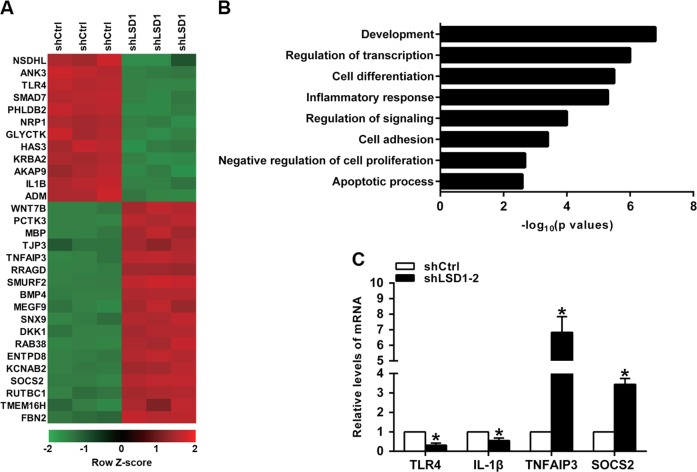
RNA-sequencing after *Lsd1* knockdown in hepatitis B virus (HBV)-infected HK-2 cells. **a** Heat map shows top 30 differentially expressed genes in shCtrl- or shLSD1-treated cells with three repeats. Differential gene expression is displayed as *Z*-score. **b** Gene ontology analysis for all genes with altered expressions. **c** The altered mRNA levels of inflammatory-related genes were selectively confirmed by quantitative real-time PCR in knockdown *Lsd1*. Data are presented as the mean ± SD (*N* = 3). *P < 0.05 versus shCtrl

To identify the target genes regulated by LSD1 in HBV-infected HK-2 cells, RNA transcriptome sequencing (RNA-seq) was carried out in controls or shRNAs. There were altogether 983 mRNAs revealed ≥1.5-fold increased abundance; conversely, 557 genes exhibited a decline in abundance (≤1.5-fold) owing to the silencing *Lsd1* (Fig. 4a and Supplementary Table S2). Gene ontology analysis revealed that many of these genes are involved in inflammatory response processes (Fig. 4b). In order to prioritize most *Lsd1*-related genes, attention was given to the genes that were most extensively expressed with knockdown of *Lsd1*. As expected, among the most highly expressed genes, many renowned genes associated with inflammation (e.g., *Tlr4*, *Il-1B*, *Tnfaip3*, and *Socs2*, et al.) are included. Verification of these inflammatory genes was carried out by qRT-PCR after *Lsd1* knockdown in HBV-infected HK-2 cells (Fig. 4c). Among these genes, one membrane receptor protein, TLR4, has increasingly been shown to play a critical role in inflammation, and it has previously been found to significantly upregulated in the kidneys of HBV-GN. Altogether, these data suggested that *Tlr4*, a downstream target of LSD1, might play an important role in regulating the expression of proinflammatory mediators in HBV-infected HK-2 cells.

### *Tlr4* is directly regulated by LSD1 in HBV-infected HK-2 cells

**Fig. 5:**
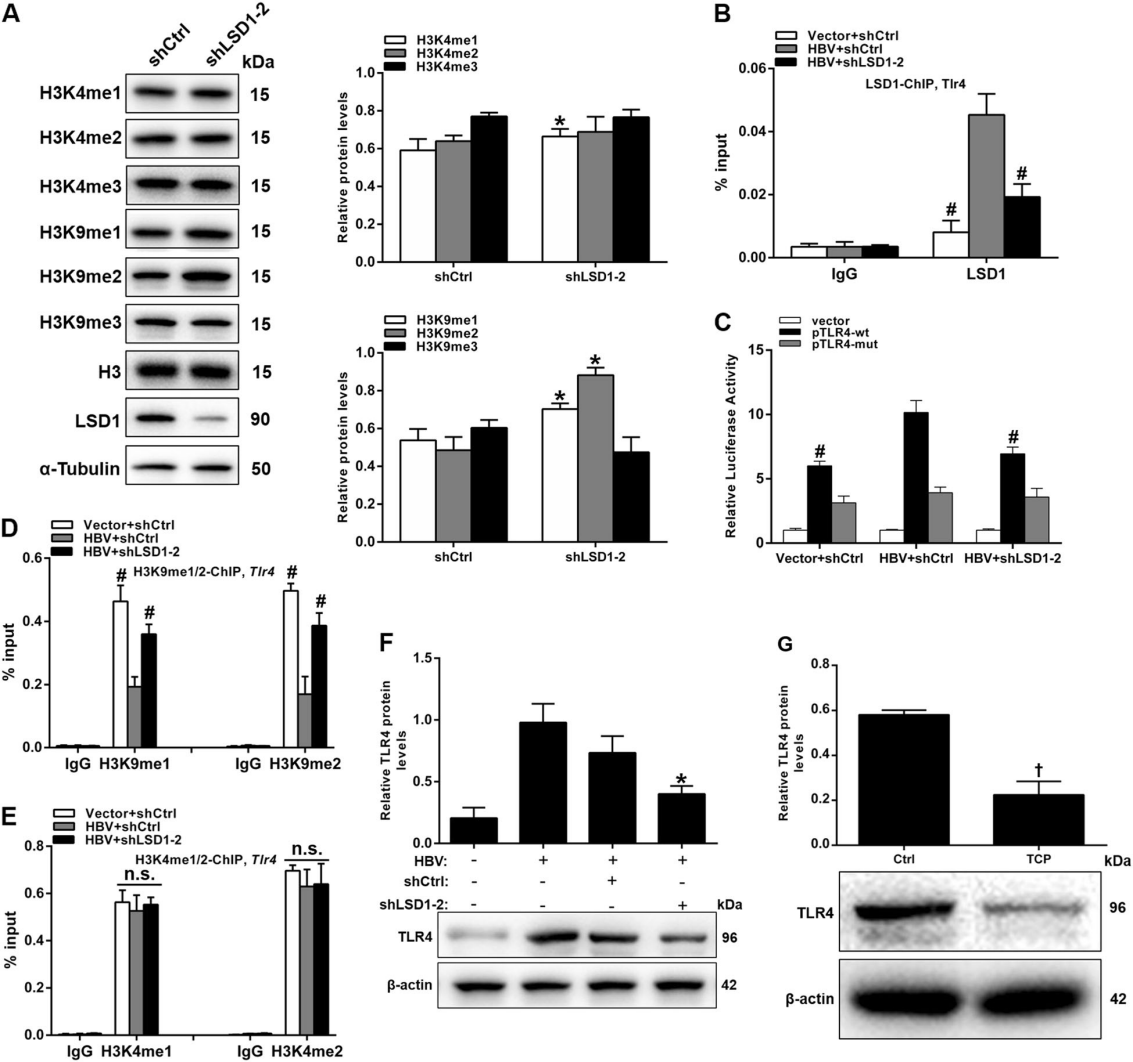
*Lsd1* directly regulates *Tlr4* expression in hepatitis B virus (HBV)-infected HK-2 cells. **a** Western blot analysis of H3K4 and H3K9 methylation in HK-2 cells co-transfected with pCMV-HBV1.3 and shCtrl or shLSD1-2 for 48 h. **b**, **d**, **e** Chromatin immunoprecipitation-quantitative PCR analysis of LSD1 (**b**), H3K9me1/2 (**d**), and H3K4me1/2 (**e**) enrichment in the *Tlr4* promoter regions. Signals are shown as a percentage of the input. IgG immunoglobulin G. **c** Luciferase activity in lysates of HK-2 cells transfected with luciferase reporter plasmids of pGL3-basic vector (vector), toll-like receptor 4 (TLR4) promoter (pTLR4-wt), or TLR4 promoter with mutation on the predicted LSD1-binding site (pTLR4-mut). Relative luciferase activity for each group was standardized using the value from the control cells transfected with pGL3-basic vector. **f** Western blot analysis of TLR4 in HBV-infected HK-2 cells transfected with shCtrl or shLSD1-2 for 48 h. **g** Western blot analysis of TLR4 in HBV-infected HK-2 cells treated with saline (Ctrl) or tranylcypromine (TCP, 10 µM) for 12 h. Data are presented as the mean ± SD (*N* = 3). *P < 0.05 versus shCtrl; ^#^*P* < 0.05 versus HBV + shCtrl group; ^†^*P* *<* 0.05 versus Ctrl. n.s. not significant

We next investigated the molecular mechanism by which LSD1 regulates *Tlr4* expression in vitro. LSD1 is a histone demethylase for H3K4me1/2 and H3K9me1/2^18^. Western blot analysis revealed that knockdown of *Lsd1* increased the total level of H3K9me1/2 in HBV-infected HK-2 cells (Fig. 5a), consistent with an important role for LSD1 in catalyzing demethylation of the repressive epigenetic mark H3K9me1/2 and activating target genes^21,22,38^.

To explore a direct correlation between LSD1 function and *Tlr4* expression, we next put an emphasis on examining LSD1 and H3K9me1/2 levels at the promoter of *Tlr4* in HK-2 cells by performing chromatin immunoprecipitation-quantitative PCR (ChIP-qPCR) analyses. The results showed that LSD1 was strongly recruited to the promoter region of *Tlr4*, with higher enrichment in HBV-infected HK-2 cells than in control cells (Fig. 5b). Knockdown of *Lsd1* decreased itself binding to the promoter of *Tlr4* in HBV-infected HK-2 cells (Fig. 5b). Similar results were also obtained from a dual-luciferase reporter assay (Fig. 5c). Correspondingly, the enrichment of H3K9me1/2 in the promoter region of *Tlr4* was much lower in HBV-infected HK-2 cells than in control cells (Fig. 5d). Knockdown of *Lsd1* in HBV-infected HK-2 cells resulted in a significant increase in H3K9me1/2 levels within the *Tlr4* promoter regions, without apparently altering H3K4me1/2 occupancy (Fig. 5d, e). In contrast to the ChIP signal at the *Tlr4* promoter regions, no occupancy of LSD1 was found in other inflammatory genes *Il-1B* and *Il-6* (Supplementary Figure S3). To further investigate whether TLR4 protein levels were regulated by LSD1 in vitro, we examined its expression in HBV-infected HK-2 cells following *Lsd1* knockdown or inhibition by western blot analyses. The results revealed that TLR4 protein levels were suppressed by *Lsd1* knockdown in HBV-infected HK-2 cells (Fig. 5f). Similar results were also obtained from LSD1 inhibition with its inhibitor tranylcypromine (TCP), a monoamine oxidase inhibitor (Fig. 5g). These data strongly suggested that LSD1 directly bound to the *Tlr4* promoters and regulated its expression.

### TLR4-related NF-κB and JNK pathways contribute to the production of proinflammatory mediators in HBV-infected HK-2 cells

**Fig. 6:**
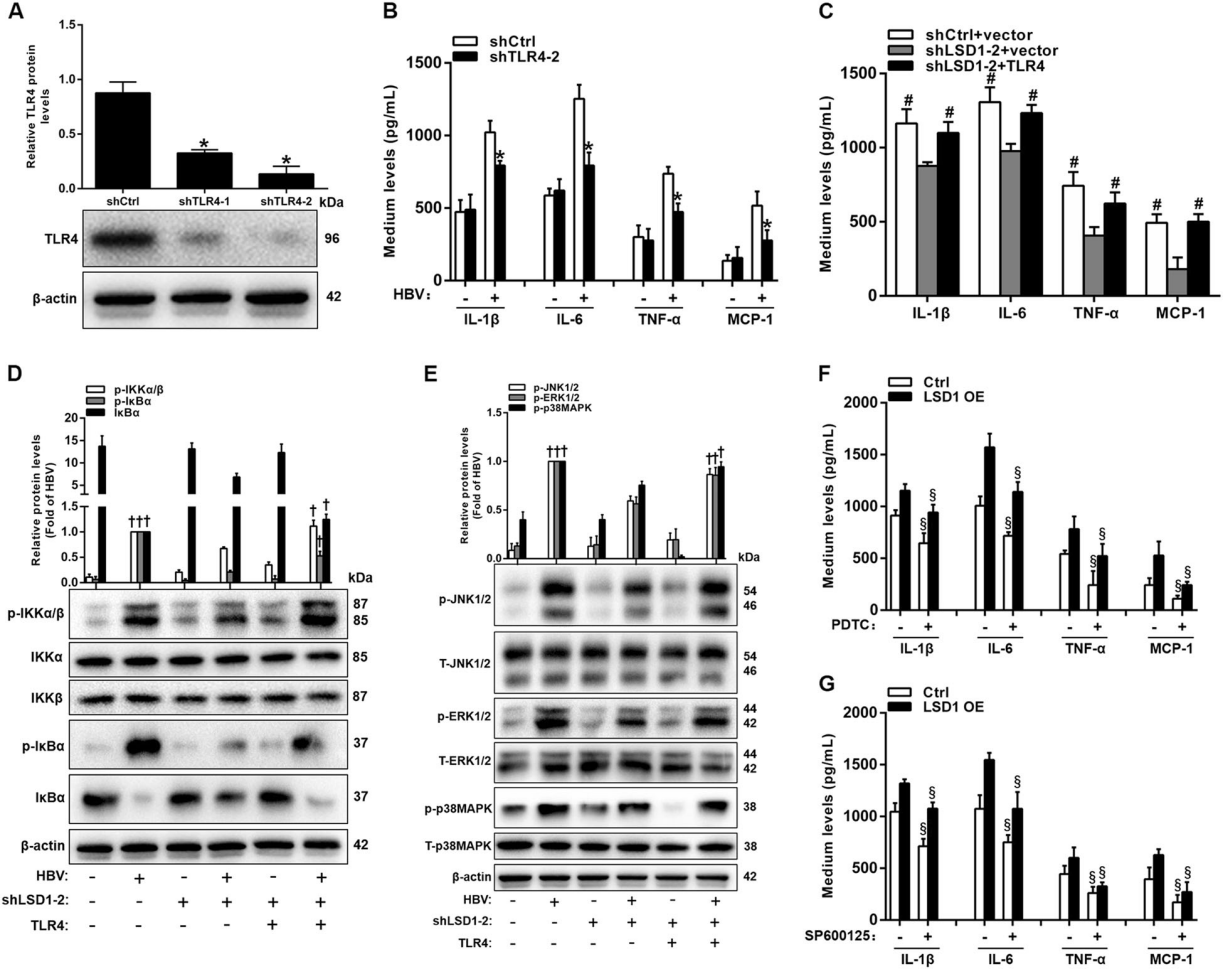
Lysine-specific demethylase 1 (LSD1) promotes the release of proinflammatory mediators in hepatitis B virus (HBV)-infected HK-2 cells via toll-like receptor 4 (TLR4). **a** Western blot analysis of TLR4 in HK-2 cells co-transfected with pCMV-HBV1.3 and shCtrl or shTLR4 for 48 h. **b** Enzyme-linked immunosorbent assay (ELISA) analysis of proinflammatory mediator levels in culture medium of HK-2 cells co-transfected with pCMV-HBV1.3 and shTLR4-2 for 48 h. **c** ELISA analysis of proinflammatory mediators levels in culture medium of HK-2 cells infected by HBV treated with shLSD1-2 along with pcDNA3.1/myc or pcDNA3.1/myc-TLR4 for 48 h. **d**, **e** Western blot analysis of p-IKKα/β, IKKα, IKKβ, p-IκBα, IκBα (**d**), p-JNK1/2, T-JNK1/2, p-ERK1/2, T-ERK1/2, p-p38MAPK, and T-p38MAPK (**e**) in HK-2 cells co-transfected with pCMV-HBV1.3 and shLSD1-2 and pcDNA3.1/myc-TLR4 for 12 h. **f**, **g** ELISA analysis of proinflammatory mediators levels in cell culture medium. HK-2 cells were pre-incubated with or without 30 µm PDTC (**f**) or 20 µm SP600125 (**g**) for 2 h and then treated with pCMV-HBV1.3 along with pcDNA3.1/myc or pcDNA3.1/myc-LSD1 for 48 h. Data are presented as the mean ± SD (*N* = 4). *P < 0.05 versus shCtrl; ^#^*P* < 0.05 versus shLSD1-2 + vector group; ^†^*P* < 0.05 versus HBV + shLSD1-2 group; ^§^*P* < 0.05 versus no PDTC or SP600125 treatment

Given that LSD1 regulates *Tlr4* expression, which is also upregulated in HBV-infected HK-2 cells (Fig. 5e), the effects of *Tlr4* knockdown on HBV-induced production of proinflammatory mediators were examined. *Tlr4* shRNAs, which could efficiently suppress TLR4 protein expression (Fig. 6a), were transfected into HK-2 cells infected with or without HBV. After 48 h of the co-transfection, the levels of proinflammatory mediators in cell culture medium were determined by ELISA assay. The results showed that *Tlr4* knockdown obviously decreased the levels of proinflammatory mediators in culture medium of HK-2 cells infected with HBV (Fig. 6b). Next, given that knockdown of *Lsd1* and *Tlr4* both abrogate HBV-induced production of proinflammatory mediators, we went on to explore whether the production of proinflammatory mediators induced by LSD1 depends on the regulation of TLR4 in HBV-infected HK-2 cells. HBV-infected HK-2 cells were simultaneously transfected with *Lsd1* shRNA and full-length *Tlr4* or control vector. The overexpression of *Tlr4* rescued the *Lsd1* knockdown-induced reduction of the release of proinflammatory mediators in culture medium of HK-2 cells infected with HBV (Fig. 6c). These results demonstrated that LSD1 promoted HBV-induced production of proinflammatory mediators in HK-2 cells via TLR4.

Considering that TLR4 has been reported to promote inflammation through NF-κB and/or MAPK pathway^39–41^, we further investigated whether LSD1 regulates HBV-induced production of proinflammatory mediators by TLR4-activaed NF-κB and/or MAPK signaling. Compared with controls, IKKα/β, IκBα, JNK1/2, ERK1/2, and p38MAPK phosphorylation had an obvious increase in HK-2 cells with HBV infection, which in turn was inhibited by *Lsd1* knockdown (Fig. 6d, e). In addition, knockdown of *Lsd1* prevented IκBα degradation (Fig. 6d). Importantly, all these effects of *Lsd1* knockdown could be counteracted by *Tlr4* overexpression in HK-2 cells. These results suggested that LSD1 could mediate the NF-κB and MAPK signaling by directly regulating *Tlr4* expression in HBV-infected HK-2 cells. Then the question was raised: could TLR4-activated NF-κB and/or MAPK signaling increase HBV-induced levels of proinflammatory mediators in HK-2 cells? To answer it, we blocked the NF-κB and MAPK signaling with the corresponding inhibitors in HBV-infected HK-2 cells (Supplementary Figure S4A–C). The results revealed that treatment with pyrrolidine dithiocarbamate (PDTC) (the NF-κΒ inhibitor) or SP600125 (the JNK inhibitor) significantly attenuated HBV- or Lsd1 overexpression-induced increase of proinflammatory mediators production in HBV-infected HK-2 cells (Fig. 6f, g). However, treatment with U0126 (the ERK inhibitor) or SB203580 (the p38MAPK inhibitor) had no effect on this process (Supplementary Figure S5A, B). Overall, these results demonstrated that LSD1 could promote HBV-induced production of proinflammatory mediators in HK-2 cells by mediating TLR4-NF-κB/JNK signaling cascades.

### LSD1 inhibitor TCP attenuates renal inflammation in HBV transgenic mice

**Fig. 7:**
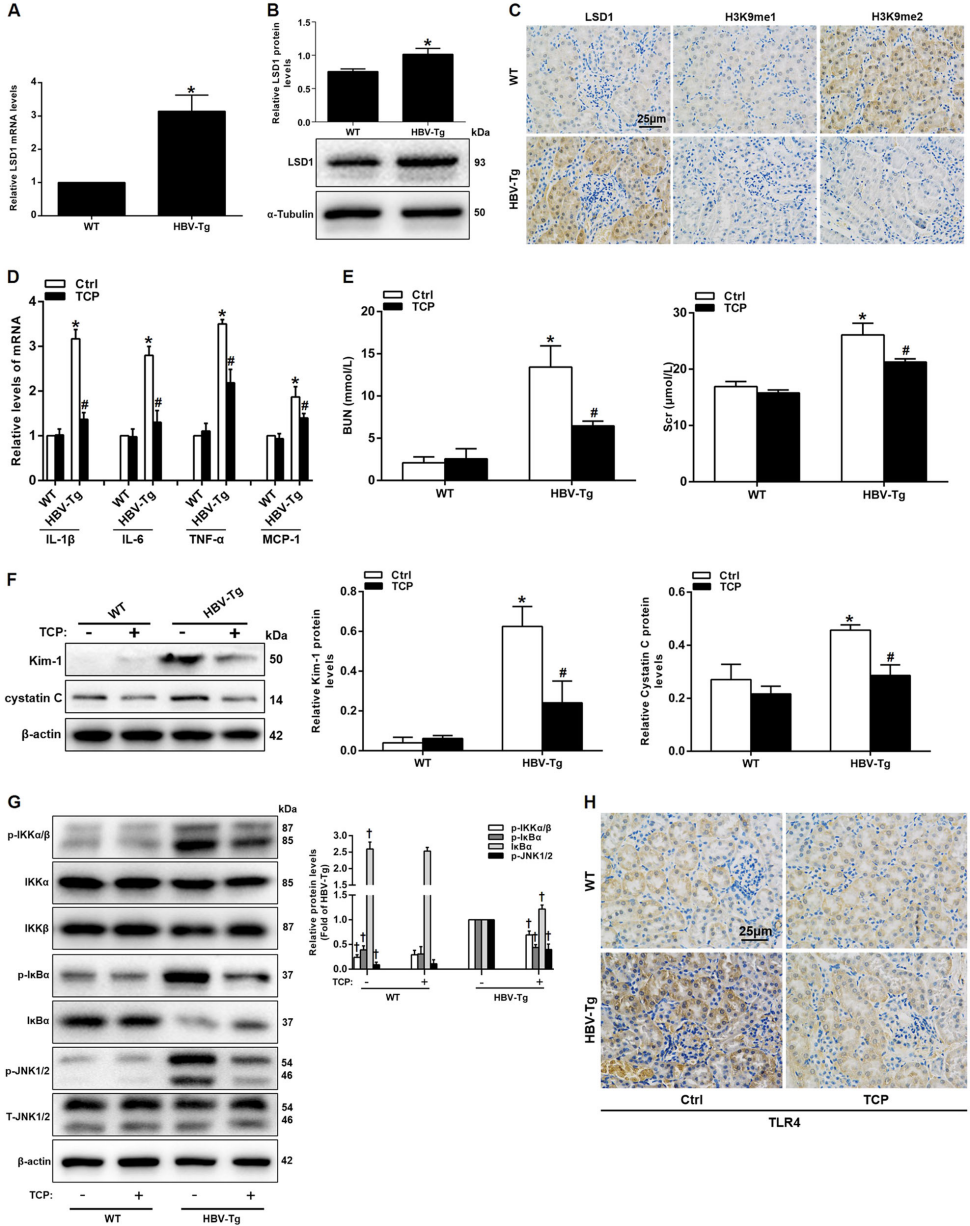
Inhibition of lysine-specific demethylase 1 (LSD1) attenuates renal inflammation in vivo. **a**, **b** Quantitative real-time PCR (qRT-PCR) and western blot analysis of LSD1 mRNA (**a**) and protein (**b**) in renal cortex of wild-type (WT) and hepatitis B virus-transgenic (HBV-Tg) mice. **c** Immunohistochemistry for LSD1 and H3K9me1/2 in the renal cortex. **d**–**h** WT or HBV-Tg mice were treated with saline or tranylcypromine (TCP). **d** qRT-PCR analysis of proinflammatory mediator mRNA levels. **e** Blood urea nitrogen (BUN) and Scr were examined by Roche Modular P800. **f** Western blot analysis of Kim-1 and cystatin C. **g** Western blot analysis of p-IKKα/β, IKKα, IKKβ, p-IκBα, IκBα, p-JNK1/2, and T-JNK1/2. **h** Immunohistochemistry for toll-like receptor 4 (TLR4) in the renal cortex. Data are presented as the mean ± SD (*N* = 3). *P < 0.05 versus WT; ^#^*P* < 0.05 versus Ctrl; ^†^*P* *<* 0.05 versus HBV-Tg alone group

To explore whether LSD1 could regulate renal inflammation in vivo, HBV transgenic (HBV-Tg) mice and wild-type (WT) C57BL/6 mice were used in the following study. In WT mice, the expression of HBsAg and HBV DNA in both serum and renal cortex was not evident. However, in HBV-Tg mice, their expression could be detected (Supplementary Figure S6A–D). The results indicated that HBV could replicate and express in the kidneys in this HBV-Tg mice. LSD1 mRNA and protein levels were upregulated in the kidneys of HBV-Tg mice compared with those of WT mice (Fig. 7a, b). IHC analysis in the renal cortex showed that LSD1 expression in RTE cells was increased in HBV-Tg mice while H3K9me1/2 expression was decreased (Fig. 7c). Meanwhile, expression levels of proinflammatory mediators were also elevated in the kidneys of HBV-Tg mice (Fig. 7d). The results suggested a possibility that the upregulation of LSD1 might contribute to renal inflammation in the kidneys of HBV-Tg mice. To confirm that, we inhibited the activity of LSD1 protein with TCP in 16-week-old WT and HBV-Tg mice. The results showed that TCP treatment significantly reduced the expression levels of IL-1β, IL-6, and TNF-α but had no significant effects on the expression levels of MCP-1 in the renal cortex of HBV-Tg mice (Fig. 7d). The serum levels of creatinine (Scr) and blood urea nitrogen (BUN) were also significantly suppressed by TCP treatment (Fig. 7e). In addition, TCP treatment could significantly reduce the protein expression levels of Kim-1 and cystatin C (Fig. 7f), which are generally used as markers of renal injury. These data indicated that TCP treatment could attenuated renal inflammation and injury in the HBV-Tg mice. Mechanistically, TCP treatment could prevent degradation of IκB-α and activation of the IKK-α/β and JNK pathways (Fig. 7g). IHC analysis in the renal cortex showed that TLR4 expression in RTE cells was increased in HBV-Tg mice, and that the upregulation of TLR4 expression was prevented by TCP treatment (Fig. 7h). Taken together, these data further demonstrated that TCP could attenuate renal inflammation and targeted inhibition of LSD1 could be a potential therapy for treating HBV-GN.

## Discussion

HBV-GN has been recognized as the most prevalent extra-hepatic lesion caused by HBV infection^42^. More and more evidences have indicated that direct HBV-induced renal inflammation plays a vital role in the progression of HBV-GN^5–8^, which may be a potential therapeutic target to mitigate the severity of HBV-GN, but the mechanisms that control its precise regulation remain obscure. In this study, we found that LSD1 was upregulated in the kidneys of HBV-GN patients compared with those of non-HBV-GN patients. Furthermore, LSD1 expression was significantly correlated with renal inflammation of HBV-GN. These data suggested that upregulation of LSD1 corresponding to HBV infection might promote the renal inflammation associated with the progression of HBV-GN.

It has been reported that LSD1 could regulate the inflammatory response in sepsis^33^ and breast cancer^36,37^, but until now little has been known about the role and molecular mechanism of LSD1 in mediating renal inflammation of HBV-GN. In this study, we found that LSD1 could promote HBV-induced renal inflammation evidenced by the increase in the expression levels of proinflammatory cytokines (IL-1β, IL-6, and TNF-α) as well as chemokines (MCP-1) in vitro. Moreover, LSD1 inhibition could attenuate renal inflammation and injury in HBV-Tg mice. These findings revealed that the crucial roles of LSD1 in renal inflammation of HBV-GN. To explore the molecular basis of LSD1 and renal inflammation, we analyzed the differential expression of the HBV-infected HK-2 cells before and after knocking down *Lsd1* by RNA-seq analysis. With the help of RNA-seq, we not only observed the fact that the findings of gene ontology analysis were associated with inflammatory response processes, but also figured out several target genes that were regulated by the LSD1. Prospectively, among these genes, four genes, including *Tlr4*, *Il-1B*, *Tnfaip3*, and *Socs2*, have been identified as inflammatory-related genes. It has also been reported that LSD1, a histone demethylase for demethylation of H3K4me1/2 or H3K9me1/2, could directly bind to the inflammatory genes *Il-1B* and *Il-6* promoters and regulate their expression^37^. Unfortunately, in our study, no occupancy of LSD1 was observed on *Il-1B* and *Il-6* promoters by ChIP-qPCR analysis in HBV-infected HK-2 cells. The results implied that LSD1 might promote these proinflammatory mediator expression by regulating other inflammatory genes or pathways rather than directly regulating their expression. As expected, our results showed that LSD1 could positively regulate the expression of *Tlr4*, which is just one of above four inflammatory genes, through catalysis of H3K9me1/2 but not H3K4me1/2 demethylation at its promoter regions in HBV-infected HK-2 cells. Consistently, previous studies have also reported that H3K9me1/2 demethylation at target promoters was observed to activate transcription^21–23,38^. In addition, our previous studies have confirmed that TLR4, which is known to activate downstream proinflammatory pathways^5,41^, was significantly upregulated in HBV-positive serum-stimulated HK-2 cells and renal biopsies from patients with HBV-GN^5^. Thus, a rational conclusion could be drawn that LSD1 might promote HBV-induced production of proinflammatory mediators by epigenetic regulation of *Tlr4*.

TLR4, a member of the TLRs, is a pattern recognition receptor that plays a crucial role in innate immunity and inflammation^43^. It has increasingly been shown to play an important role in viral infections including HBV infection^5^. It is clear that HBsAg, as the HBV membrane protein, can activate the TLR signaling and thereby promote the release of inflammatory cytokines^44^. In hepatic studies, TLR4 binding to HBV envelope proteins has also been shown to induce the expression and secretion of TNF-α, IL-12, and IL-6 and other proinflammatory factors, which exacerbates the destruction of liver tissue^44,45^. These data clearly suggest that TLR4 is of importance in inducing the inflammatory response during HBV infection. Consistently, our results also showed that *Lsd1* knockdown could markedly inhibit HBV-induced *Tlr4* expression in HK-2 cells. Moreover, *Tlr4* expression regulated by LSD1 could promote proinflammatory mediators secretion under HBV infection condition. It has also been reported that TLR4 activation by various stimuli could activate downstream proinflammatory pathways, such as the NF-κB and/or MAPK pathways, and thereby result in the release of proinflammatory cytokines and chemokines causing inflammation, which contributes to the pathogenesis of inflammation-associated renal injury^5,46,47^. Therefore, we attempted to explore whether LSD1 could activate TLR4-NF-κB/MAPK pathways to promote HBV-induced production of proinflammatory mediators.

NF-κB, an inflammatory transcription factor, has an important role in the pathogenesis of HBV-associated nephropathy^35,48^. NF-κB activation is triggered by p65 phosphorylation and IκB phosphorylation and subsequent degradation, which causes NF-κB translocation to the nucleus and subsequent transcription of several target genes^49^. The activation of NF-κB is closely linked to the physiological immunity and pathological inflammation^49,50^. TLR4 is known to promote NF-κB activation in progressive renal injury^46^. In the present study, LSD1 induced degradation of IκBα and activation of the IKKα/β through mediation of TLR4 in HBV-infected HK-2 cells. Importantly, treatment with the NF-κΒ inhibitor PDTC could attenuate the *Lsd1* overexpression-induced increase of proinflammatory mediators expression. These results indicated that LSD1-mediated TLR4-NF-κB signaling could promote HBV-stimulated production of proinflammatory mediators in vitro. In addition, another inflammatory transcriptional factor, AP-1, is mediated by MAPK families such as JNK, ERK, and p38MAPK in response to cellular inflammatory stimuli that regulate proinflammatory mediators^51–53^. TLR4 is also known to activate the MAPK pathways and produce proinflammatory cytokines, such as TNF-α, IL-1β, and IL-6^54^. Consistent with these findings, our results revealed that LSD1 also induced phosphorylation of JNK, ERK, and p38MAPK by TLR4 in HBV-infected HK-2 cells. However, following inhibition with the corresponding inhibitors, only the JNK inhibitor SP600125 significantly reduced the production of proinflammatory mediators induced by *Lsd1* overexpression by mediation of *Tlr4* in HBV-infected HK-2 cells. These findings suggested that LSD1-mediated TLR4 downstream JNK signaling but not ERK and p38MAPK signaling promoted HBV-induced production of proinflammatory mediators in HK-2 cells. At last, pretreatment with LSD1 inhibitor TCP in HBV-Tg mice could downregulate TLR4 expression and subsequently suppress degradation of IκBα and phosphorylation of IKKα/β, IκBα, and JNK, thereby possibly inhibiting the expression of proinflammatory mediators. Taken together, these data demonstrated that TLR4-NF-κB/JNK signaling axis regulated by LSD1 might promote HBV-induced renal inflammation in vitro and in vivo.

The limitations of this study are also noted. First, the molecular mechanism by which LSD1 epigenetically regulated *Tlr4* expression was investigated only in vitro, but not in vivo. Similarly, the NF-κΒ and MAPK pathway inhibitors were used only in vitro, and more investigations for their roles should be provided in vivo in future studies. In addition, the component of HBV that could activate TLR4 to promote HBV-induced renal inflammation was not demonstrated in this study.

**Fig. 8:**
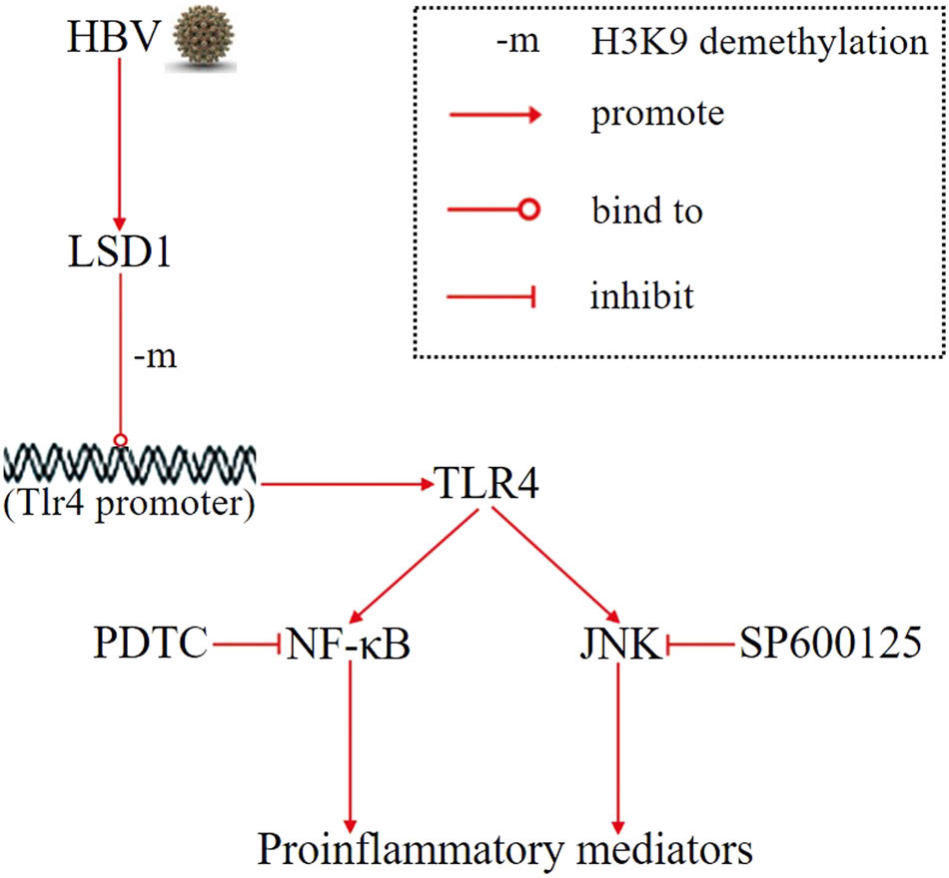
The proposed mechanism of lysine-specific demethylase 1 (LSD1) involved in promoting hepatitis B virus (HBV)-induced production of proinflammatory mediators via toll-like receptor 4 (TLR4). LSD1 promotes HBV-induced production of proinflammatory mediators in vitro by epigenetically upregulating TLR4, thereby contributing to the activation of nuclear factor-κB (NF-κB) and JNK pathways

In summary, this study demonstrated that LSD1 was upregulated in renal tissue of HBV-GN. LSD1 could promote HBV-induced renal inflammation in human RTE cells by epigenetic regulation of the TLR4 signaling pathways. LSD1 inhibition could attenuate inflammation in the renal cortex of HBV-Tg mice (Fig. 8) Therefore, LSD1 might be a potential target in the intervention of HBV-GN progression.

## Materials and methods

### Patient specimens

We collected renal biopsy specimens in paraffin blocks that had been diagnosed as HBV-GN at the Department of Nephrology of Shanghai Jiaotong University Affiliated Hospitals during 2015 and 2017. The diagnosis of HBV-GN was made by serological detection of HBV antigens and antibodies, and immunohistochemical demonstration of HBV-related antigens as well as immune complexes in a kidney biopsy specimen^55^. According to the pathological type and serum HBV antigens, we divided samples into four groups: group I, the HBV-GN group (both renal tissue and serum HBsAg positive), 53 samples; group II, the HBV-positive PGN group (PGN patients with serum HBsAg or HBV DNA positive) including 35 samples; group III, the HBV-negative PGN group (PGN patients without HBV infection), including 50 samples; and group IV, normal control group (15 samples from the adjacent normal renal tissues of tumor resection specimens). Moreover, groups II–IV were collectively called non-HBV-GN groups. The clinical and histologic patient characteristics are presented in Supplementary Table S1. The study was approved by the Ethics Committee of Shanghai General Hospital, Shanghai Jiaotong University School of Medicine, and conducted in accordance with ethical principles of the World Medical Association Declaration of Helsinki and local legislation. Appropriate consent was obtained from all patients.

### Immunofluorescence

The 4-μm formalin-fixed paraffin-embedded renal tissue sections were dewaxed and placed into water. After antigen repairing, the tissue sections were blocked with 5% bovine serum albumin and then incubated with HBsAg and E-cadherin antibody (Abcam, Cambridge, MA, UK) overnight at 4 °C. After three times washes with phosphate-buffered saline, secondary antibody was added and incubated for 2 h at room temperature (RT), and then stained with 4′,6-diamidino-2-phenylindole (Invitrogen) for 2 min. Images were captured using a fluorescence microscope (Olympus BX51TF, Tokyo, Japan).

### Immunohistochemistry

Adjacent 4-μm formalin-fixed paraffin-embedded human and mouse kidney sections were immunohistochemically stained for LSD1, IL-6, MCP-1, CD68, H3K9me1/2, TLR4 (Abcam, Cambridge, MA, USA), and IL-1β (Cell Signaling Technology, Danvers, MA, USA) protein expression with an avidin-biotin-peroxidase complex method. Pictures were taken under a light microscope (DP70, Olympus, Tokyo, Japan). Then, immunohistochemical staining results were evaluated and scored as reported previously^6^.

### Plasmids and reagents

The plasmid pCMV-HBV1.3, which expresses HBV (genotype C, serotype adr), was obtained from GenePharma Co., Ltd (Shanghai, China). The plasmid pcDNA3.1/myc-LSD1 or pcDNA3.1/myc-TLR4 was constructed by inserting a PCR-cloned *Lsd1*-gene cDNA or *Tlr4*-gene cDNA into pcDNA3.1/myc-His(−)B vectors (Invitrogen, Carlsbad, CA, USA) and was then verified by sequencing. LSD1-specific shRNAs, shLSD1-1 and shLSD1-2, and TLR4-specific shRNAs, shTLR4-1 and shTLR4-2, were cloned into pGPU6/GFP/Neo vector (GenePharma Co., Ltd, Shanghai, China), the shRNA target sequences are listed in Supplementary Table S3. The pGL3-basic luciferase vector (Promega, Madison, WI, USA) was used to clone the promoter of TLR4, and LSD1-binding site-specific mutant was generated by PCR. Thus, pGL3-TLR4-wt and pGL3-TLR4-mut plasmids were separately constructed. TCP hydrochloride was purchased from Sigma-Aldrich (St Louis, MO, USA). Chemistry inhibitors of NF-κB (PDTC), JNK (SP600125), ERK (U0126), and p38MAPK (SB203580) were obtained from Beyotime (Shanghai, China).

### Cell culture and transfection

HK-2 cells, a human proximal tubule cell line, were obtained from American Type Culture Collection (Rockville, MD, USA). The cells were cultured in DMEM/F12 medium containing 10% fetal bovine serum and 1% penicillin/streptomycin (Gibco, NY, USA) in a humidified atmosphere of 5% CO_2_ at 37 °C, and plasmids were transfected into the cells with Lipofectamine 3000 (Invitrogen, Carlsbad, CA, USA).

### Western blot analysis

Total proteins from the samples were separated by 6–12% SDS-polyacrylamide gel electrophoresis and then transferred onto 0.22 μm polyvinylidene difluoride membrane (Millipore, Billerica, MA, USA). Antibodies against LSD1, TLR4, and Kim-1 were purchased from Abcam (Cambridge, MA, USA). Antibodies against H3, H3K4me1, H3K4me2, and H3K4me3 were purchased from ABclonal Technology (Boston, MA, USA). Antibodies against H3K9me1, H3K9me2, H3K9me3, p-IKKα/β, IKKα, IKKβ, IκBα, p-IκBα, p-JNK1/2, JNK1/2, p-ERK1/2, ERK1/2, p-p38MAPK, and p38MAPK were purchased from Cell Signaling Technology (Danvers, MA, USA). Antibody against Cystatin C was purchased from Bioworld Technology, Ltd (Nanjing, Jiangsu, China). Antibodies against α-Tubulin and β-actin were purchased from Santa Cruz Biotechnology (Santa Cruz, CA, USA).

### Quantitative real-time PCR

Total RNAs were extracted using Trizol reagent (Invitrogen, Carlsbad, CA, USA) and complementary DNA was synthesized with Reverse Transcription system (Toyobo, Osaka, Japan) according to the manufacturer’s instructions. Real-time PCR was performed on a Light-Cycler (Roche Diagnostics, Mannheim, Germany) using SYBR Green Premix (Takara, Otsu, Japan). The expression of target genes was normalized to the expression of GAPDH. The data were analyzed by delta Ct method. The primer sequences used in this study are listed in Supplementary Table S3.

### Enzyme-linked immunosorbent assay

The levels of IL-1β, IL-6, TNF-α, and MCP-1 in the supernatants were determined using ELISA kits (eBioscience, San Diego, CA, USA). The HBsAg and HBeAg levels in the supernatants or serum were also determined by ELISA kits (R&D, Minneapolis, MN, USA).

### HBV DNA analysis

Mice sera were collected, and an HBV fluorescence quantitative detection kit (Da-An, Guangzhou, China) was used according to the instructions. The copies of HBV DNA were quantified using qRT-PCR as described previously^56^.

### RNA-sequencing

Total RNA was isolated using TRIZol (Invitrogen) from the HBV-infected HK-2 cells transfected with shCtrl or shLSD1. A total of 250 ng of each sample was used to prepare libraries using a TruSeq Stranded mRNA Sample Prep Kit (Illumina, San Diego, CA, USA) according to the manufacturer’s instructions. Sequencing was performed using an Illumina HiSeq 2500. Raw pair-end reads were mapped to the human genome (hg38) with Tophat2 (v2.1.1). Differentially expressed genes were generated with Cuffdiff (v1.3.0) with false discovery rate value < 0.05 and filtered by corresponding threshold. Data can be accessed in Supplementary Table S2.

### Chromatin immunoprecipitation

ChIP assay was performed using the SimpleCHIP enzymatic chromatin immunoprecipitation kit (Cell Signaling Technology, MA, USA) as described previously^35^. After treatment, the cells were harvested and crosslinked with 1% (v/v) formaldehyde for 10 min at RT. The protein-bound immunoprecipitated DNA and DNA isolation protocols were performed as described earlier^57^. Thereinto, antibodies against LSD1 (Abcam, Cambridge, MA, USA), H3K4me1/2, H3K9me1/2, and immunoglobulin G (Cell Signaling Technology, Danvers, MA, USA) were used in this study. The ChIP DNA was purified with PCR purification kit (Qiagen) and quantified by real-time PCR The ChIP-PCR primers are listed in Supplementary Table S3.

### Dual-luciferase reporter assay

HK-2 cells were seeded into 24-well plates and transfected. Briefly, the pGL3-TLR4-wt and pGL3-TLR4-mut plasmids were separately co-transfected with the pRL-TK renilla vector using Lipofectamine 3000 (Invitrogen). Twelve hours after transfection, cells were co-transfected with pCMV-HBV1.3 and LSD1 shRNAs for another 24 h. Cells were harvested, and luciferase activity was measured as described previously^35^. The ratio of firefly luciferase to renilla luciferase was calculated in each group.

### Mice

Male HBV-Tg C57BL/6 mice aged 6 weeks were purchased from Vitalstar Biotechnology Co., Ltd. (Beijing, China). Male WT C57BL/6 mice were obtained from the same source and served as controls. Animals were maintained under specific pathogen-free conditions at 22 °C under a 12 h light/dark cycle. We measured 24 h serum creatinine (Scr) and BUN with an automatic biochemical analyzer. After mice were sacrificed, kidneys were collected for IHC and western blot analysis. All experiments were approved by the Animal Care and Use Committee of Shanghai General Hospital, Shanghai Jiaotong University School of Medicine, and were performed in accordance with the US National Institutes of Health Guide (NIH publication, Eighth edition, 2011) for the Care and Use of Laboratory Animals.

### Statistical analysis

Statistical analysis was performed using the GraphPad Prism 6 (GraphPad Software, Inc., Jolla, CA, USA). The data were expressed as the mean ± SD values and analyzed by Student’s *t*-test, *χ*^2^ test, or one-way analysis of variance. The correlation analyses were analyzed by the Spearman rank-order correlation. *P*-values < 0.05 were considered statistically significant.

## Supplementary information

Supplementary Figures

Supplementary Tables
