# Supplementary material for: The histone demethylase LSD1 promotes renal inflammation by mediating TLR4 signaling in hepatitis B virus-associated glomerulonephritis
Source: Cell Death Dis. 2019 Mar 20;10(4):278. doi: 10.1038/s41419-019-1514-4 (PMC6427019; doi:10.1038/s41419-019-1514-4)
Supplement: Supplementary file 1 — Supplementary Figures [file 41419_2019_1514_MOESM1_ESM.docx]

**
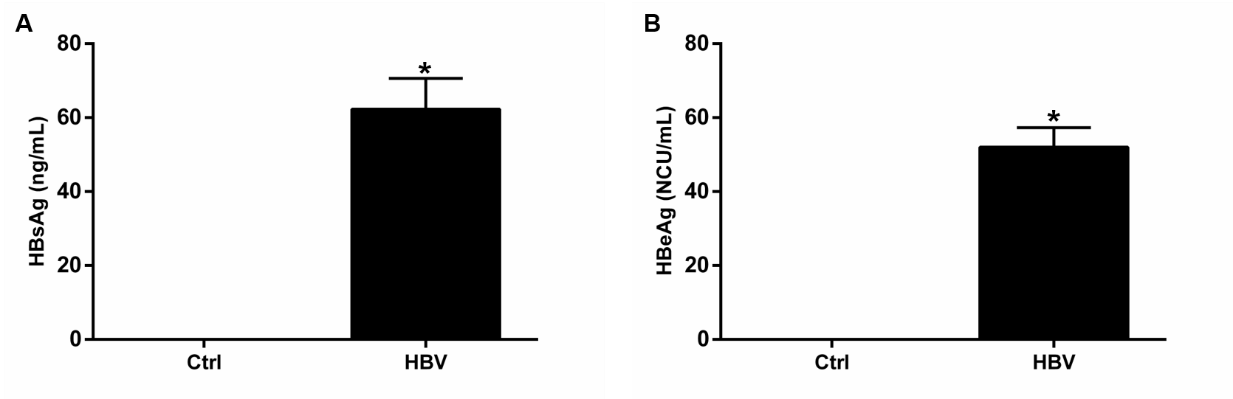
**

**Supplementary Figure 1** Levels of HBsAg and HBeAg in the cell supernatant. **A, B** HK-2 cells were transfected with a control (Ctrl) or pCMV-HBV1.3 plasmid for 48 h. The levels of HBsAg (**A**) and HBeAg (**B**) were detected by ELISA. Data were presented as mean ± SD (N = 3). **P* < 0.05 versus Ctrl

**
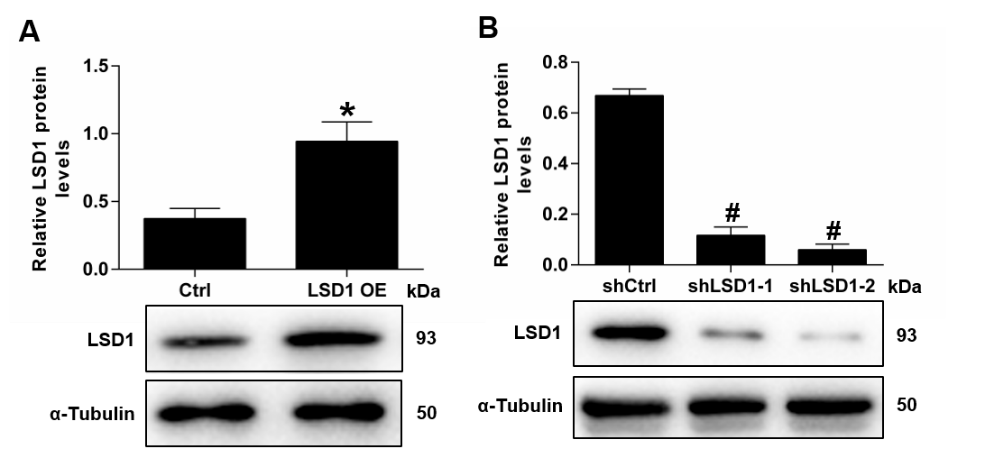
**

**Supplementary Figure 2** LSD1 overexpression and knockdown in HBV-infected HK-2 cells. **A, B** HK-2 cells were transfected with pCMV-HBV1.3 along with pcDNA3.1/myc (Ctrl) or pcDNA3.1/myc-LSD1 (LSD1 OE) (**A**) or with control shRNA (shCtrl) or LSD1 shRNAs (shLSD1) (**B**) for 48 h. LSD1 protein levels were determined by western blot. Data are presented as the mean ± SD (N = 3). **P* < 0.05 versus Ctrl; ^#^*P* < 0.05 versus shCtrl

**
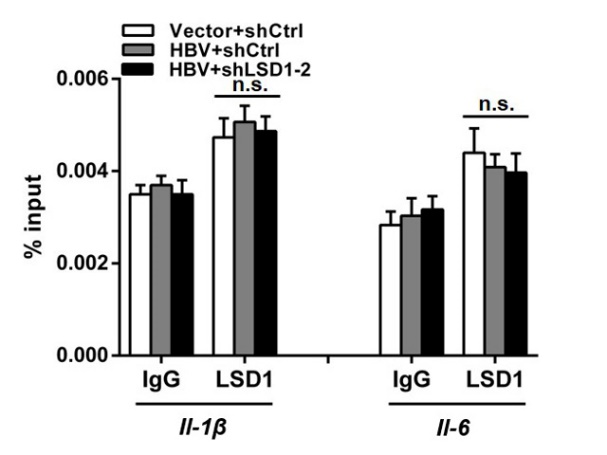
**

**Supplementary Figure 3** LSD1 is not significantly recruited to the promoter region of *Il-1B and Il-6* in HBV-infected HK-2 cells. HK-2 cells with HBV infection were transfected with shLSD1-2. ChIP-qPCR analysis of LSD1 enrichment in the *Tlr4* promoter regions. Signals are shown as a percentage of the input. IgG, immunoglobulin G. Data are presented as the mean ± SD (N = 3). n.s., not significant

**
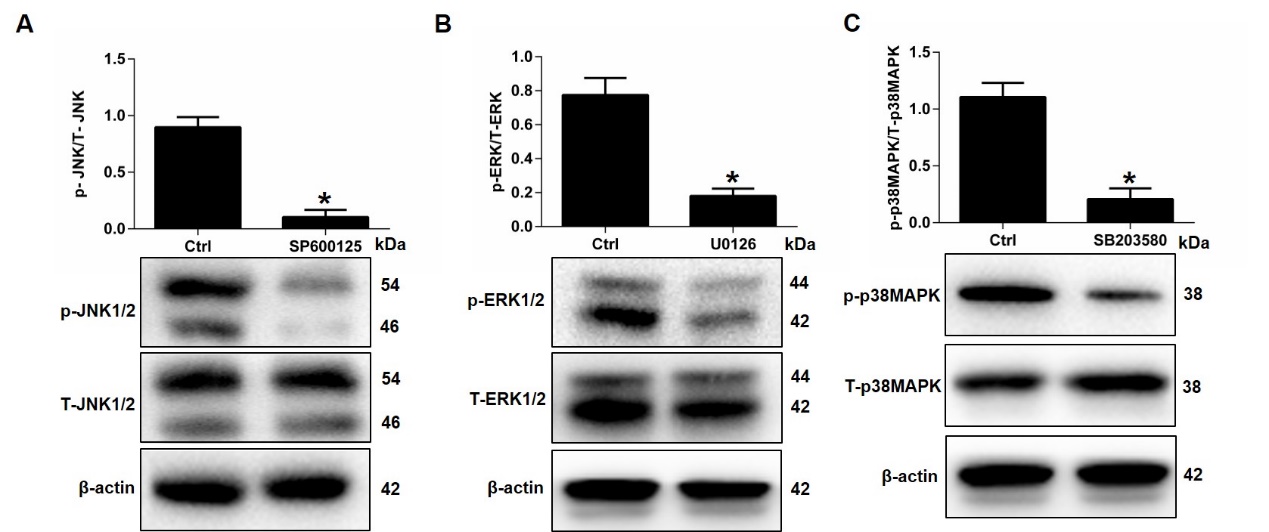
**

**Supplementary Figure 4** MAPKs specific inhibitors can effectively suppress the corresponding pathways. **A**-**C** HK-2 cells were pre-incubated with or without 20 µm SP600125 (**A**), 20 µm U0126 (**B**), or 10 µm SB203580 (**C**) for 2 h and then treated with pCMV-HBV1.3 for 24 h. The expression levels of p-JNK1/2, T-JNK1/2, p-ERK1/2, T-ERK1/2, p-p38MAPK, and T-p38MAPK were determined by western blot. Data are presented as the mean ± SD (N = 3). **P* < 0.05 versus Ctrl

**
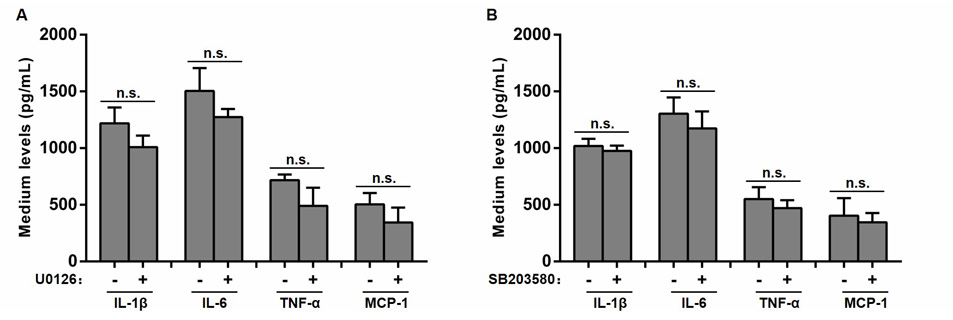
**

**Supplementary Figure 5** Both ERK inhibitor U0126 and P38 inhibitor SB203580 cannot suppress the release of proinflammatory mediators in HBV-infected HK-2 cells. **A, B** HK-2 cells were pre-incubated with or without 20 µm U0126 (**A**) or 10 µm SB203580 (**B**) for 2 h and then treated with pCMV-HBV1.3 for 48 h. IL-1β, IL-6, TNF-α, and MCP-1 levels were detected by ELISA. Data are presented as the mean ± SD (N = 3). n.s., not significant

**
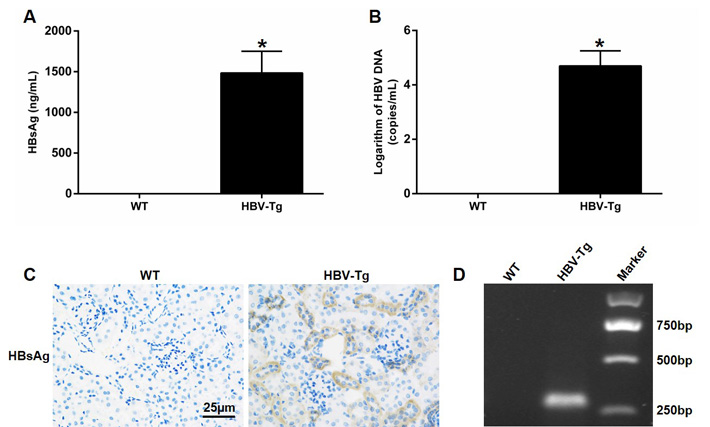
**

**Supplementary Figure 6** Expression of HBsAg and HBV DNA in serum and renal cortex of WT and HBV-Tg mice. **A, B** The serum levels of HBsAg and HBV DNA were detected by ELISA (**A**) and FQ-PCR (**B**), respectively. **C** Immunohistochemistry analysis of HBsAg in the renal cortex. **D** RT-PCR analysis of HBV DNA in the renal cortex. Data are presented as the mean ± SD (N = 3). **P* < 0.05 versus WT
