# Supplementary material for: The histone demethylase LSD1 promotes renal inflammation by mediating TLR4 signaling in hepatitis B virus-associated glomerulonephritis
Source: Cell Death Dis. 2019 Mar 20;10(4):278. doi: 10.1038/s41419-019-1514-4 (PMC6427019; doi:10.1038/s41419-019-1514-4)
Supplement: Supplementary file 2 — Supplementary Tables [file 41419_2019_1514_MOESM2_ESM.docx]

**Supplementary Table S1** Clinical manifestations, pathology, virology and liver function in the studied subjects

| **Item** | **Group I (n = 53)** | **Group II (n = 35)** | **Group III (n = 50)** |
| --- | --- | --- | --- |
| Average age (years) | 39 | 40 | 37 |
| Male-to-female ratio | 35:18 | 21:14 | 33:17 |
| Hematuria cases (%) | 26 (49.1%) | 17 (48.5%) | 24 (48.0%) |
| Edema cases (%) | 33 (62.3%) | 20 (57.1%) | 29 (58.0%) |
| SBP (mmHg) | 137.48 ± 6.53 | 133.16 ± 6.72 | 134.05 ± 6.20 |
| DBP (mmHg) | 90.06 ± 3.51 | 88.24 ± 4.01 | 86.31 ± 4.82 |
| Urinary protein (g) | 2.42 ± 0.21 | 2.26 ± 0.33 | 2.23± 0.27 |
| CCr (mL/min 1.73 m2) | 94.4 ± 10.11 | 91.7 ± 9.8 | 92.3 ± 10.5 |
| hs-CRP (mg/L) | 2.61 ± 1.21 | 2.51 ± 1.38 | 2.44 ± 1.57 |
| Glomerular score | 2.68 ± 1.40 | 2.55 ± 1.35 | 2.71± 1.42 |
| Tubular score | 3.39 ± 2.13 | 3.09 ± 2.07 | 2.99 ± 3.02 |
| HBsAg (+) | 53 (100%) | 35 (100%) | / |
| HBcAg (+) | 22 (41.5%) | 14 (40.0%) | / |
| HBV DNA (> 10^3^ copies/mL) | 26 (49.1%) | 17 (48.6%) | / |
| ALT (U/L) | 34 ± 4.43 | 33 ± 3.45 | 31 ± 3.22 |
| AST (U/L) | 28 ± 6.02 | 29 ± 5.49 | 28 ± 5.31 |

**Supplementary Table S2** Top 30 differentially expressed genes in HBV-infected HK-2 cells before and after knocking down LSD1

| **Gene symbol** | **Description** | **log_2_FC** | **FDR** | **Style** |
| --- | --- | --- | --- | --- |
| NSDHL | NAD(P) dependent steroid dehydrogenase-like | -2.451 | 0.000 | down |
| ANK3 | Ankyrin-3, node of ranvier | -2.122 | 0.000 | down |
| TLR4 | Toll like receptor 4 | -2.097 | 0.000 | down |
| SMAD7 | SMAD family member 7 | -2.065 | 0.000 | down |
| PHLDB2 | Pleckstrin homology like domain family B member 2 | -1.996 | 0.000 | down |
| NRP1 | Neuropilin 1 | -1.954 | 0.000 | down |
| GLYCTK | Glycerate kinase | -1.875 | 0.000 | down |
| HAS3 | Hyaluronan synthase 3 | -1.833 | 0.000 | down |
| KRBA2 | KRAB-A domain containing 2 | -1.793 | 0.000 | down |
| AKAP9 | A-Kinase anchoring protein 9 | -1.738 | 0.000 | down |
| IL1B | Interleukin 1 beta | -1.720 | 0.000 | down |
| ADM | Adrenomedullin | -1.709 | 0.000 | down |
| WNT7B | Wnt family member 7B | 5.562 | 0.000 | up |
| PCTK3 | PCTAIRE protein kinase 3 | 5.284 | 0.000 | up |
| MBP | Myelin basic protein | 4.867 | 0.000 | up |
| TJP3 | Tight junction protein 3 | 3.752 | 0.000 | up |
| TNFAIP3 | TNF alpha induced protein 3 | 3.616 | 0.000 | up |
| RRAGD | Ras related GTP binding D | 3.581 | 0.000 | up |
| SMURF2 | SMAD specific E3 ubiquitin protein ligase 2 | 3.296 | 0.000 | up |
| BMP4 | Bone morphogenetic protein 4 | 2.815 | 0.000 | up |
| MEGF9 | Multiple EGF like domains 9 | 2.173 | 0.000 | up |
| SNX9 | Sorting nexin 9 | 2.159 | 0.000 | up |
| DKK1 | Dickkopf WNT signaling pathway inhibitor 1 | 2.072 | 0.000 | up |
| RAB38 | RAB38, member RAS oncogene family | 2.002 | 0.000 | up |
| ENTPD8 | Ectonucleoside triphosphate diphosphohy-drolase 8 | 1.824 | 0.000 | up |
| KCNAB2 | K(+) Channel Subunit Beta-2 | 1.778 | 0.000 | up |
| SOCS2 | Suppressor of cytokine signaling 2 | 1.749 | 0.000 | up |
| RUTBC1 | RUN And TBC1 domain-containing protein 1 | 1.711 | 0.000 | up |
| TMEM16H | Transmembrane protein 16H | 1.703 | 0.000 | up |
| FBN2 | Fibrillin 2 | 1.680 | 0.000 | up |

**Supplementary Table S3** Primer sequences and shRNA target sequences are listed below

| **Molecule** | **Primer** | **Sequence** |
| --- | --- | --- |
| Human LSD1 | forward | 5’-TCCTGGCCCCTCGATTC-3’ |
|  | reverse | 5’-ATGTTCTCCCGCAAAGAAGAGT-3’ |
| Mouse LSD1 | forward | 5’-ATGGATGTCACACTTCTGGA-3’ |
|  | reverse | 5’-CAAGACCTGTTACAACCATG-3’ |
| Human IL-1β | forward | 5’-ATGATGGCTTATTACAGTGGCAA-3’ |
|  | reverse | 5’-GTCGGAGATTCGTAGCTGGA-3’ |
| Mouse IL-1β | forward | 5’-GAAAGACGGCACACCCACCCT-3’ |
|  | reverse | 5’-GCTCTGCTTGTGAGGTGCTGATGTA-3’ |
| Human IL-6 | forward | 5’-ACTCACCTCTTCAGAACGAATTG-3’ |
|  | reverse | 5’-CCATCTTTGGAAGGTTCAGGTTG-3’ |
| Mouse IL-6 | forward | 5’-TCCAGTTGCCTTCTTGGGAC-3’ |
|  | reverse | 5’-GTGTAATTAAGCCTCCGACTTG-3’ |
| Human TNF-α | forward | 5’-CCTCTCTCTAATCAGCCCTCTG-3’ |
|  | reverse | 5’-GAGGACCTGGGAGTAGATGAG-3’ |
| Mouse TNF-α | forward | 5’-CGTCAGCCGATTTGCTATCT-3’ |
|  | reverse | 5’-CGGACTCCGCAAAGTCTAAG-3’ |
| Human MCP-1 | forward | 5’-ACTCTCGCCTCCAGCATGAA-3’ |
|  | reverse | 5’-TTGATTGCATCTGGCTGAGC-3’ |
| Mouse MCP-1 | forward | 5’-CCTCCACCACTATGCAGGTC-3’ |
|  | reverse | 5’-CAGCCGACTCATTGGGATCA-3’ |
| Human TLR4 | forward | 5’-GCTTATCTGAAGGTGTTGCA-3’ |
|  | reverse | 5’-CAGAGTTTCCTGCAATGGAT-3’ |
| Human TNFAIP3 | forward | 5’-ATACCCCATTGTTCTCGGCTAT-3’ |
|  | reverse | 5’-AATCTTCCCCGGTCTCTGTTAA-3’ |
| Human SOCS2 | forward | 5’-CAGATGTGCAAGGATAAGCGG-3’ |
|  | reverse | 5’-GCGGTTTGGTCAGATAAAGGTG-3’ |
| Human GAPDH | forward | 5’-TGGTATCGTGGAAGGACTCATGAC-3’ |
|  | reverse | 5’-ATGCCAGTGAGCTTCCCGTTCAGC-3’ |
| Mouse GAPDH | forward | 5’-CATGACCACAGTCCATGCCATCAC-3’ |
|  | reverse | 5’-TGAGGTCCACCACCCTGTTGCTGT-3’ |
| Human TLR4 promoter (ChIP) | forward | 5’-GTGAGTTTCTTCACAAGAAGGG-3’ |
|  | reverse | 5’-GGAGAGAGAGCCTTGAAAGAGG-3’ |
| Human IL-1β promoter (ChIP) | forward | 5’-AAAGCTTGGTGATGTCTGGTC-3’ |
|  | reverse | 5’-GGACATGGAGAACACCACTTG-3’ |
| Human IL-6 promoter (ChIP) | forward | 5’-TTCAATGAGGAGACTTGCCTGGTGA-3’ |
|  | reverse | 5’-TCTGCACAGCTCTGGCTTGGTTCC-3’ |
| Human shLSD1-1 | / | 5’-TACATCTTACCTTAGTCATCT-3’ |
| Human shLSD1-2 | / | 5’-TGAAGGCTTGGACATTAAACT-3’ |
| Human shTLR4-1 | / | 5’-GCATAGAGGTACTTCCTAATA-3’ |
| Human shTLR4-2 | / | 5’-GTACATGTGGATCTTTCTTAT-3’ |
